# An australian audit of vaccination status in children and adolescents with inflammatory bowel disease

**DOI:** 10.1186/1471-230X-11-87

**Published:** 2011-07-29

**Authors:** Nigel W Crawford, Anthony G Catto-Smith, Mark R Oliver, Donald JS Cameron, Jim P Buttery

**Affiliations:** 1 SAEFVIC, Department of General Medicine, Royal Children's Hospital (RCH), Melbourne, Victoria 3011 Australia; 2 Murdoch Childrens Research Institute (MCRI), Melbourne, Victoria 3011 Australia; 3 Department of Paediatrics, The University of Melbourne, Victoria 3011 Australia; 4 Department of Gastroenterology & Clinical Nutrition, RCH, Melbourne, Victoria 3011 Australia; 5 Gastroenterology Unit, Monash Children's Hospital, Southern Health, Melbourne, Victoria 3011 Australia; 6 Paediatric Infectious Diseases Unit, Monash Children's Hospital, Southern Health, Melbourne, Victoria 3011 Australia; 7 Department of Paediatrics, Monash University, Melbourne, Victoria 3011 Australia

**Keywords:** pediatric, infections, inflammatory bowel disease, immunosuppressed, immunization

## Abstract

**Background:**

Children and adolescents with inflammatory bowel disease (IBD) are at increased risk of vaccine preventable diseases (VPD). This includes invasive pneumococcal disease and influenza. The primary aim of this study was to describe compliance with current Australian guidelines for vaccination of children and adolescents diagnosed with IBD. A secondary aim was to review the serological screening for VPD.

**Methods:**

A random sample of patients (0-18 years at diagnosis), were selected from the Victoria Australia state based Pediatric Inflammatory Bowel Disease Register. A multi-faceted retrospective review of immunization status was undertaken, with hospital records audited, a telephone interview survey conducted with consenting parents and the vaccination history was checked against the primary care physician and Australian Childhood Immunization Register (ACIR) records. The routine primary childhood vaccinations and administration of the recommended additional influenza and pneumococcal vaccines was clarified.

**Results:**

This 2007 audit reviewed the immunization status of 101individuals on the Victorian Pediatric IBD database. Median age at diagnosis was 12.1 years, 50% were on active immunosuppressive therapy. 90% (38/42) [95% confidence intervals (CI) 77%; 97%] with complete immunization information were up-to-date with routine primary immunizations. Only 5% (5/101) [95% CI 2%; 11%] received a recommended pneumococcal vaccine booster and 10% (10/101) [95% CI 5%; 17%] had evidence of having ever received a seasonal influenza vaccine. Those living in rural Victoria (p = 0.005) and younger at the age of diagnosis (p = 0.002) were more likely to have ever received an influenza vaccine Serological testing, reviewing historical protection from VPD, identified 18% (17/94) with evidence of at least one serology sample.

**Conclusion:**

This study highlights poor compliance in IBD patients for additional recommended vaccines. A multi-faceted approach is required to maximize protection from VPD in this vulnerable special risk population.

## Background

The prevention of vaccine preventable diseases (VPD) in all individuals with inflammatory bowel disease (IBD) is increasingly recognised as important[1-3]. IBD rates have increased globally over the past 30 years, including in the Australian state of Victoria, where the incidence of Crohn's disease rose from 0.128 to 2.0 per 100 000 per year over a 31-year period (1971-2001)[4]. Immunosuppressive therapies such as corticosteroids and azathioprine have long been used in IBD management. Infliximab is one of the biologic anti-TNF-alpha antibody therapies, and an effective treatment for luminal and fistulising Crohn's disease, as well as those with a high inflammatory disease burden[5].

The risk of opportunistic infections in IBD is increased, particularly when on immunosuppressive therapies, including biologics such as infliximab[6,7]. This risk is increased when combining two or more immunosuppressive medications[8,9]. There are case reports of VPD in this vulnerable group, including severe varicella and pneumococcal pneumonia[10,11]. Viral hepatitis is also a significant risk as shown in a number of studies[12-14]. Cheveuax et al. found evidence of seroprotection from hepatitis B through immunization in 48.9% of IBD patients (N = 315 patients), with age at diagnosis (> 31 years), duration of disease over 7 years and a diagnosis of Crohn's disease, all associated with the lack of effective vaccination. It is important to consider screening for opportunistic infections as part of the clinical approach to IBD patients and maximise prevention of these infections where possible through immunization[15]. This has been detailed in a number of guidelines, including the European evidence based consensus on the prevention, diagnosis and management of opportunistic infections in inflammatory bowel disease[16,17].

In the state of Victoria, Australia, pediatric gastroenterologists care for the majority of children and adolescents with IBD. This is predominantly at the states two tertiary gastroenterology centres, The Royal Children's Hospital (RCH) and Monash Medical Centre (MMC), Melbourne. This management in a small number of patients is shared care with a general paediatrician, particularly for those patients living in rural areas. As there is centralised service delivery, Victoria has established a state-based IBD register for children and adolescents with IBD[4].

Vaccinations in early childhood are captured in a hand held person medical record and also on the Australian Childhood Immunization Register (ACIR) for those < 7 years of age. At the time of the study ACIR data was not accessible once a child was greater than 7 years. The Australian routine funded vaccines on the National Immunization Program are detailed in Table 1. Annual influenza immunization is also funded for special risk groups such as children and adolescents with IBD ≥ 6 months of age and vaccination offered from late February through to October. Many immunization guidelines, including those in Australia, are also now recommending additional pneumococcal vaccine for special risk patients, including those with IBD on immunosuppressive medication[18]. The treating gastroenterologist is often the main source of immunization advice in childhood and adolescent IBD, particularly if immunosuppressive therapy is commenced. At RCH there is an Immunization Drop-in Centre open business hours, staffed by nurse immunization specialists, where queries can be directed and vaccines administered on the day of clinic appointments. In Victoria, immunization nurse specialist or primary care physicians administer the majority of vaccines.

**Table 1:** Australian National Immunization Program (NIP) Schedule

Age	Australian Routine Schedule Vaccines by Antigen
**Birth**	Hepatitis B
**2 months**	Diphtheria
	Tetanus
	Pertussis (acellular)
	Polio
	Haemophilus Influenzae type b (*Hib*)
	Hepatitis B
	7-valent pneumococcal conjugate vaccine* (7vPCV)
	Rotavirus^†^
	
**4 months**	Diphtheria
	Tetanus
	Pertussis
	Polio
	Hib
	Hepatitis B
	7vPCV
	Rotavirus
**6 months**	Diphtheria
	Tetanus
	Pertussis
	Polio
	Hib
	Hepatitis B
	7vPCV
	Rotavirus
**12 months**	Measles
	Mumps
	Rubella
	Hib
	Meningococcal C
**18 months**	Varicella**
**4 years**	Diphtheria
	Tetanus
	Pertussis
	Polio
	Measles
	Mumps
	Rubella
**12-13 Years**	Quadrivalent Human Papillomavirus (HPV^††^) vaccine
	Varicella and Hepatitis B vaccine catch-up if required
**15-16 years**	Diphtheria
	Tetanus
	Pertussis (acellular) [dTap 'booster']

*pneumococcal conjugate vaccine (PCV7) commenced January 2005 (catch-up for children born after January 2003)

^† ^Rotavirus vaccine program commenced June 2007

** Varicella vaccine on routine NIP schedule November 2005

^†† ^HPV vaccine on NIP schedule April 2007- females only, catch-up 12-26 year olds 2007-09

The primary aim of this study was to describe the compliance with current Australian guidelines for vaccination of children and adolescents diagnosed with IBD current at the time of the study[19]. A secondary aim was to review the serological screening for VPD in patients with IBD.

## Methods

A multi-faceted retrospective review of immunization status was undertaken, with the inclusion criteria being age 0-18 years at diagnosis and on the IBD register. There were a total of nine gastroenterologists working across the two tertiary units [RCH and MMC] at the time of the study. Hospital records of all participants were audited, with any vaccinations administered recorded in the outpatient notes and/or a medication chart if administered at the RCH Immunization Drop-in-centre. A telephone interview survey was conducted with consenting parents using the parent-held child immunization record. The vaccination history was checked against the primary care physician and ACIR records. The routine primary childhood vaccinations and administration of the recommended additional influenza and pneumococcal vaccines was clarified. Therapies were categorized into four groups: ASA derivatives (sulphasalazine, osalazine, mesalazine and balsalazide); oral corticosteroids (prednisolone); immunosuppressive agents (azathioprine, methotrexate) and biologics (infliximab). The RCH patient's hospital laboratory results were reviewed to identify if any baseline serological testing was performed to review the requirement for additional protection against VPD such as varicella and hepatitis B. Multiple sources were reviewed for any vaccine safety concerns or reports of adverse events following immunization, including: hospital records, general practitioner records and parent reports through interviews.

A random sample of 101 participants was taken from the IBD register. The random sample was generated using the statistical software STATA Version 10.0 (StataCorp, TX), which was also used for data analysis. This overall sample size calculation was based on the outcome of routine immunization up-to-date status by hospital medical record audit and allowed a determination of proportions within +/- 10% with 95% confidence. Proportions of up-to-date status and additional vaccines administered were compared using a Pearson chi-square test with point estimate odds ratios (OR) and 95% confidence intervals determined and a p value < 0.05 considered statistically significant. The study was approved by the Royal Children's Hospital Human Research Ethics Committee.

## Results

The study was conducted between July-November 2007. The participant demographics are detailed in Table 2. There only difference at baseline between those selected for the study, compared with the rest of the IBD database (N = 534), was proportionally more cases of indeterminate colitis in the database group [11% versus 3%; P = 0.01]. Participants had a median age at diagnosis of 12.1 years [range 1.9 to 17.8 years], 50% were female, and 75% had Crohn's disease. At the time of the immunization audit 43 participants were on 5-ASA derivatives: sulphasalazine (17), mesalazine (21), besalazine (4) and osalazine (1). Reviewing ongoing immunosuppressive therapies, there were 39 participants, some on multiple medications including: oral corticosteroids (7), azathioprine (36) and infliximab (7).

**Table 2:** Baseline Characteristics

Characteristic	Audit (%)	IBD Database (%)	P value
Sex (Female)	50	41	0.12
			
Diagnosis			
Crohn's disease	74	70	0.4
Ulcerative colitis	23	19	0.4
Indeterminate colitis	3	11	0.01
Length time since diagnosis (range: years)			
≤ 5years	64	55	0.12
≥6 years	36	45	0.12
			
Medication exposure (ever)*			
Oral steroids (> 3 months duration)	77		
Antiinflammatory (sulphasalazine/osalazine/mesalazine)	88		
Immunosuppressive (azothioprine or methotrexate)	59		
Biologic therapy (Anti TNF Infliximab)	20		

* some patients had multiple medications so percentages may exceed 100%

**Figure 1:**
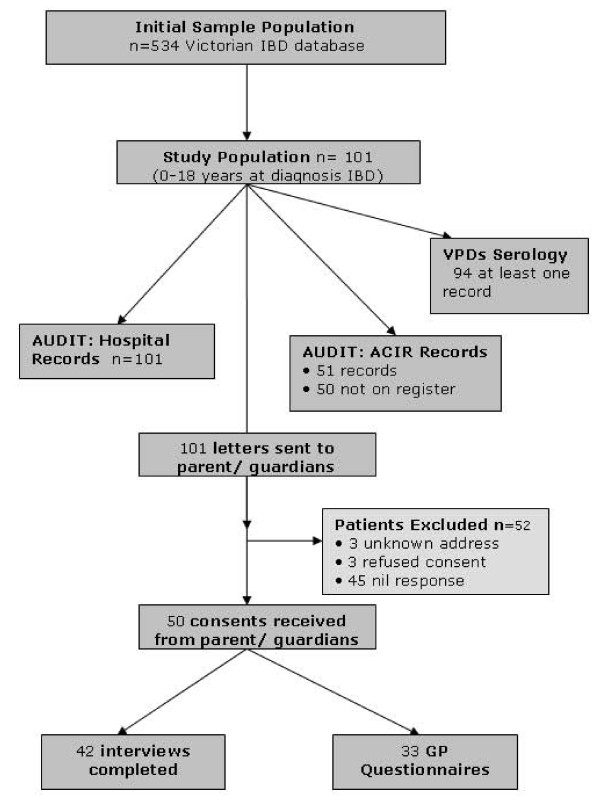
**Flow diagram of study participants**.

One hundred and one hospital patient records were reviewed as detailed in the study flow diagram. (Figure 1) A telephone immunization survey was completed in 42% and primary care practitioner records obtained in 66% (33/50) of consenting participants. In those whom a complete telephone immunization survey was obtained, 90% (38/42) [95% confidence intervals (CI) 77%; 97%] were up-to date with routine primary childhood immunizations.

For additional recommended vaccines, only 5% (5/101) [95% CI 2%; 11%] had received a recommended pneumococcal 'booster' and all were on active therapy including azathioprine (4) and infliximab (1). 10% (10/101) [95% CI 5%; 17%] had evidence of having ever received an influenza vaccination, 7% (7/101) [95% CI 3%; 14%] in the year of the survey. Those living in rural Victoria (Odds ratio 6.51 95% CI 1.33; 41.25, p = 0.005) and younger at the age of diagnosis (Pearson square for trend χ^2 ^= 16.8; p = 0.002) were more likely to have received an influenza vaccine. The reasons for not having received an influenza vaccine (n = 33) included: not being aware of it (24%); concerned about side effects (24%) not necessary (15%); doctor did not offer it (6%), allergy (6%) and unspecified or other reason (25%).

Serological testing, reviewing historical protection from VPD, identified 18% (17/94) with evidence of at least one serology sample. Having serology testing was not associated with age (χ^2 ^for trend = 1.7; p = 0.42). For the patients who had varicella serology, 18% (2/11) were negative. Pre-diagnosis varicella vaccine had been received by 8 participants, with an additional 30 confirming pre-diagnosis clinical infection. There were four participants (none currently on infliximab) who had hepatitis B serology documented, all were negative for hepatitis B surface antigen (HepBsAg) and seropositive for protective anti-HepB surface antibody. On Hepatitis B vaccination history, immunization was confirmed in 93% (39/42), including 86% (6/7) of those currently on infliximab. The quadrivalent human papillomavirus (4vHPV) vaccine had been administered to 14 young women. There were no documented safety concerns or flares of inflammatory bowel disease notified following immunization.

## Discussion

This study highlights good compliance with routine childhood immunizations, with the 90% up-to date status equivalent to the coverage rates of 93.6% at seen in Victoria, Australia overall[20]. The uptake of recommended additional vaccines such as annual influenza vaccination and pneumococcal vaccine boosters was low.

There are very few published studies on compliance guidelines on immunizations in IBD patients[2,21]. In a United States immunization status survey of 169 adolescents and adults diagnosed with IBD, 86% were using immunosuppressive therapies[22]. On immunization status recall only 28% had received influenza and 13% pneumococcal vaccination. This compares with this study of influenza vaccination confirmed in 10% and additional pneumococcal vaccination 5%. When reviewing other special risk groups' compliance with recommended additional vaccines, a Victorian study of childhood cancer survivors found 47% had 'ever' had an influenza vaccine[23]. This compared to 27% of children < 7 years diagnosed with cystic fibrosis and managed at RCH who had received an annual influenza vaccine and 37% the additional recommended pneumococcal vaccine[24]. In addition in a study of preterm infants at two Victorian tertiary neonatal units, only 20% of infants with chronic lung disease had received an influenza vaccine[25].

The increased risk of morbidity and mortality from influenza infection in those with a chronic illness such as IBD is well described and confirmed in the 2009-10 HINI influenza epidemic[26,27]. The response to influenza vaccine in patients receiving immunomodulatory therapy is variable, but usually satisfactory, as seen in an influenza vaccine sero-response study in 146 IBD patients, including those who were immunosuppressed[28]. The safety of influenza vaccine in IBD patients has been reviewed in a cohort of adult IBD patients following H1N1 influenza vaccination [29]. Four weeks after immunization, absence of a disease flare was observed in 96.7% patients with Crohn's disease and 95.6% with ulcerative colitis. The importance of physician recommendation for uptake of influenza vaccine, has been highlighted in a parental survey of children with a chronic illness[30].

Invasive pneumococcal disease (IPD) is also increased in those with an underlying chronic illness and on immunosuppressive therapies[31]. It is hence important to protect IBD patients from these VPD. Australia introduced a 7-valent conjugate pneumococcal vaccine (PCV7) on 1 Jan 2005, and the special risk guidelines current in 2007 recommended additional 7vPCV if under 10 years and 23 valent polysaccharide vaccine 23vPPV if > 10 years of age[18]. The response in adults to 23vPPV is variable and IBD patients on biologic therapies such as infliximab achieve lower protective titres[32]. Newer pneumococcal conjugate vaccines with increasing numbers of serotypes are becoming available internationally [33] and the 13 valent conjugate vaccine (PCV13) introduced into a number of countries including the United States and soon in Australia[34,35]. Importantly the PCV13 vaccine is also being studied in adults > 50 years, and early results from phase III immunogenicity studies found an equivalent or better response than the 23vPPV[36]. Studies are also in progress to determine the efficacy of this vaccine in preventing community acquired pneumonia in adults > 65 years[37]. Studies of these newer PCV are required in special risk populations to assess both their safety and immunogenicity and help determine the best schedule to optimise IPD protection.

The risk of reactivation of diseases such as hepatitis B by these anti-TNFα antibody therapies, has led to recommendations of additional screening before commencing therapy[38,39]. In this study 20% of participants had been treated with infliximab and other biologic therapies may be used in the future. Reassuringly, 93% (39/42) of those with comprehensive immunization records had documented evidence of hepatitis B vaccination. However, serological testing confirming a negative Hepatitis B infection status was conducted in only four patients. It is possible this is an underestimate as serology samples may have been sent to external laboratories. Another limitation of this serological evaluation was that it was conducted at only one of the two sites (RCH), but included 94% of subjects.

Live vaccines need to be considered with caution in patients who are immunosuppressed, due to the risk of vaccine associated morbidity such as seen with measles vaccine[40]. Varicella vaccine was introduced into Australian on 1 November 2005 for all children aged 18 months with a catch-up program at age 12-13 years. In this study 9% (4/42) were found to not have a history of clinical infection or immunization against varicella placing them at risk, similar to the 10% found in the United States IBD survey[22]. The morbidity of varicella in immunosuppressed patients has led to studies challenging the general recommendation that this live attenuated vaccines not be administered[41]. A case series of six young people with IBD safely receiving varicella vaccination whilst on infliximab therapy[42]. Despite small numbers of patients, it highlights the need to discuss the risk versus benefits of live attenuated vaccines on a case-by-case basis. Another approach is to review the immunization status at diagnosis and give all recommended vaccines (e.g. varicella, hepatitis B) before commencing immunosuppressive therapy. This, however, is becoming a smaller window of opportunity with the earlier use of immunomodulatory therapy in IBD.

A limitation of the study is the retrospective nature of the review in a state based IBD population. It was a representative sample of the database, but some IBD patients may have been excluded if seeing a private and/or adult gastroenterologist and not placed on the register. One major difficulty in conducting the study was uptake of 'opt in' consent, reflected by only 53% of adolescent and young adult patients being contactable. These are common issues in retrospective health related research and a number of surveys have explored the importance of exploring meaningful non-consent and considering 'opt out' consent[43,44]. The study was powered for 100 audit participants, but the results were affected by the low recruitment for the telephone immunization status review. The reported proportions of participants receiving the additional pneumococcal and influenza vaccines may not reflect the actual immunization status due to this incomplete record obtainment. The study was also underpowered to detect differences in the characteristics of those who received influenza vaccine. Another limitation is that the ACIR only routinely captures and maintains immunization records in children < 7 years of age, so added little information in this predominantly adolescent population. Whilst ACIR can include additional vaccines (e.g. influenza and pneumococcal), this information is not always captured.

Suboptimal compliance with annual influenza vaccination and other additional vaccines recommended could be due to lack of awareness or acceptance of immunization guidelines for IBD patients. Engagement with subspecialists like pediatric gastroenterologists managing these complex patients is important. As the primary source of healthcare related advice for IBD patients, surveying their opinions regarding immunization would be helpful and lack of this information is a study limitation. An Australian survey of adult gastroenterologists found hepatitis B, influenza and pneumococcal vaccines were recommended infrequently and the window before significant immunosuppressive therapies commenced not always being utilised[45].

Strategies to optimise protection from VPD include education of both patients and their parents/carers, as highlighted by the reasons given for influenza vaccination not being received. Education itself needs to be supported by system changes and this may include immunization reminders, which in the form of cards, telephone and electronic, have all been shown to be effective[46,47]. Protecting IBD patients through "cocooning", by ensuring parents and siblings are protected against VPD (e.g. influenza, pertussis and varicella), is also recommended.

## Conclusion

This study highlights a high level of vaccination coverage with routine scheduled vaccinations, but poor compliance with current guidelines for influenza and pneumococcal vaccination in adolescents and children with IBD. Improving serological assessment prior to commencing immunosuppressive therapies can help minimise the risk of reactivation of VPD such as hepatitis B. An approach using both direct and indirect protective immunization strategies is required to maximise protection from vaccine preventable diseases in this vulnerable population.

## Conflict of interests disclosure

NWC has investigator-led study support for a study of Guillain-Barre Syndrome Surveillance post H1N1 influenza vaccination [CSL] and been on a Pfizer [Wyeth] advisory board for pneumococcal vaccines and presented at conferences, for which his MCRI research fund has received honoraria. AGC has chaired an advisory board for GSK - rotavirus vaccine and chaired an advisory board for MSD - Infliximab in Crohn's disease. JPB sits on a data safety monitoring board for influenza vaccines [CSL] and MCRI has received conference travel reimbursement from GSK for Rotavirus vaccine conference presentation. MRO and DJSC have no conflicts of interests to declare.

## Authors' contributions

NWC and JPB conceived the study concept. All authors contributed to the study design and audit questionnaire. TCS provided the Victorian IBD database information. NWC undertook the statistical analysis and wrote the initial draft. All authors contributed to the draft review and have read and approved the final manuscript.

## Pre-publication history

The pre-publication history for this paper can be accessed here:

http://www.biomedcentral.com/1471-230X/11/87/prepub
